# Warning on False or True Morels and Button Mushrooms with Potential Toxicity Linked to Hydrazinic Toxins: An Update

**DOI:** 10.3390/toxins12080482

**Published:** 2020-07-29

**Authors:** Emmeline Lagrange, Jean-Paul Vernoux

**Affiliations:** 1 EFSN, Pôle de Neurologie, CHUGA, Grenoble University Hospital, 38000 Grenoble, France; ELagrange@chu-grenoble.fr; 2 Unité de Recherche Aliments Bioprocédés Toxicologie Environnements (ABTE) EA 4651, Normandie University, UNICAEN, 14000 Caen, France

**Keywords:** gyromitrin, true morels, button mushroom, agaritine, safe consumption

## Abstract

Recently, consumption of the gyromitrin-containing neurotoxic mushroom *Gyromitra* sp. (false morel), as gourmet food was hypothesized to play a role in sporadic amyotrophic lateral sclerosis genesis. The present review analyses recent data on edibility and toxicity of false and true morels and *Agaricus* spp. Controversy about the toxic status of *Gyromitra esculenta* was due to variable toxin susceptibility within consumers. We suggest that *Verpa bohemica*, another false morel, is also inedible. We found a temporary neurological syndrome (NS) with cerebellar signs associated with high consumption of fresh or dried true morels *Morchella* sp. After ingestion of crude or poorly cooked fresh or dried morels, a gastrointestinal “haemolytic” syndrome was also observed. Agaritine, a water soluble hydrazinic toxin closely related to gyromitrin is present along with metabolites including diazonium ions and free radicals, in *Agaricus* spp. and *A. bisporus,* the button mushroom, and in mice after ingestion. It is a potential weak carcinogen in mice, but although no data are available for humans, a lifetime low cumulative extra cancer risk in humans can be estimated to be about 10^−5^. To conclude, a safety measure is to avoid consuming any true morels or button mushrooms when crude or poorly cooked, fresh or dried.

## 1. Introduction

Mushrooms are the fruiting bodies of macroscopic filamentous fungi that grow generally above the ground. They are members of the kingdom of Fungi, which is constituted of a core of clades collectively deemed “True fungi” or Eumycota [1,2]. Using the advent of genomics and phylogenomics, a recent update reported 9 major phylum-level clades for true fungi [3]. Some species with fruiting bodies, such as morels, Helvella and truffles, along with filamentous fungi producing mycotoxins [4], belongs to Ascomycota phylum. All the other macroscopic mushroom species belong to the Basidiomycota phylum. Mushrooms fruiting bodies are used as ubiquitous components of human food and also have a medicinal use [5]. However, a fraction of known mushroom species are hazardous, and toxic substances are also found in some mushroom species with beneficial properties [6]. Even if some species have an edible status, low concentration of toxins can be present, and either chronic ingestion or over-consumption can impact the edibility status of such mushroom species. As a consequence, macroscopic mushrooms are divided into true edible, conditionally edible or poisonous categories [7]. For the conditionally edible category, edibility is linked to the ability to eliminate potential toxins or substances by cooking or drying, soaking and other pre-treatments. A list of common mushroom poisonings was given recently [7,8]. One of the main problems of mushroom food poisoning is also misidentification of toxic species during harvesting [7,9]. Species identification is possible through DNA-based molecular techniques [10,11], but for common pickers, it is based on “phenotypical characters”. Such characters can be easily recognized by a mushroom expert or a mycologist but not always by a layperson.

It was recently pointed out that, due to the gyromitrin toxin, false morels *Gyromitra* spp. could play a role in sporadic amyotrophic lateral sclerosis (ALS), a neurodegenerative disease [12,13]. Gyromitrin is a hazardous neurotoxic hydrazine derivative [14]. Furthermore, false morels can be confused with true morels, *Morchella* spp., for which delimitation of species remains complex because of their high morphological stasis and plasticity of apothecium colour and shape [15]. This makes the differentiation with related species such as false morels, more difficult. Therefore, it is of public health interest to consider the toxicity of *Gyromitra* close-species such as other false morels or true morels and also of mushroom species having hydrazinic toxins closely related to gyromitrin like *Agaricus* spp. Furthermore, the consumption of these species is very significant in France and in Europe. *Morchella esculenta* and *Agaricus campestris* are two of the five top-selling edible fungi and are listed in most European edible mushroom lists; *Agaricus* is one of the 3 genera with most species authorised for commercialisation [16]. *A. bisporus* is widely cultivated to produce low cost mushroom fruiting bodies and is the basis of a multibillion-dollar world industry. The main producer of the more costly morels is China, and its annual export of dried morels increased fivefold from 181,000 kg to 900,000 kg from 2010 to 2015, averaging $160 US dollars per kilogram [17]. Field cultivation of morels expanded to a large scale in China in recent years to around 500 tons of fresh morels per year [18]. Therefore, possible exposure to associated toxic compounds through ingestion of these mushroom species could concern a lot of people, especially regular and/or high mushroom consumers, in mycophilic countries in Europe [16]. The present paper is an updated analysis of edibility and toxicity of false and true morels and of *Agaricus* spp., especially button mushroom.

## 2. False and True Morels and Reported Toxic Effects

### 2.1. Poisonings from Crude, Cooked or Dried Gyromitra sp. or Verpa bohemica

**Figure 1:**
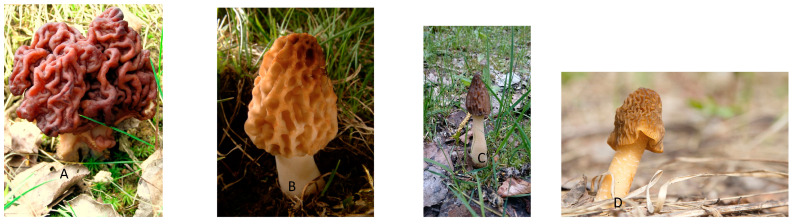
Pictures of some False and True morels. (**A**) The False morel *Gyromitra esculenta* which has a wrinkled, brain-like cap. Source: Wikimedia Commons (public domain). Photographer: Severine Meibner. (**B**) A true yellow morel, *Morchella esculenta,* which has a honeycomb cap attached at its basis to the stipe. Source: Wikimedia Commons (Public domain). Photographer: J.Marqua. (**C**) *Morchella semilibera,* a true black morel *(=Mitrophora semilibera).* Source: Wikimedia Commons (Public domain). Photographer: Abaddon1337. (**D**) The false morel *Verpa bohemica,* which has a wrinkled and subdimpled cap, put like a thimble over a finger and without any attachment at its basis to the stipe. Source: Wikimedia Commons (Public domain). Photographer Tatiana Bulyonkova. For species recognition, colour being variable, specific morphological traits is a basis for differentiation between *Gyromitra* sp. and *Morchella* sp. At the contrary, *Verpa bohemica* and *Morchella semilibera,* have similar traits.

Typical false morels are *Gyromitra* spp., which does not belong to the Morchellaceae family (Figure 1A); they are highly toxic gyromitrin-containing species [9,19,20]. Ingestion of *G. esculenta* which may be misidentified with true morel *M. esculenta,* results in early-onset gastrointestinal toxicity (GIT) from its principal toxin, gyromitrin, within 4 to 6 h. This subacute onset of gastrointestinal toxicity (nausea, vomiting, abdominal pain) may rarely be followed by epileptogenic neurotoxicity such as vertigo, delirium, seizures, stupor and coma [9]. This results from the hepatic phase 1 activation (hydrolysis) of gyromitrin to monomethylhydrazine (MMH). MMH destroys pyridoxine, a glutamic acid decarboxylase cofactor, which diminishes production of γ-amino butyric acid (GABA), a central inhibitory neurotransmitter. A similar description of this syndrome and its progressive development in two successive steps, a GIT one and then a neurotoxic one, was recently given [8]. This is reminiscent of a dose-effect linked to amount absorbed and/or toxin metabolites availability. In fact, for this last point, the metabolic hepatic status of detoxication plays a role, and either fast or low acetylators can be distinguished among consumers [8,19,21]. Therefore, for *Gyromitra esculenta* consumers, the poisoning impact, especially chronic poisoning, could be effectively mitigated when they have a fast acetylator hepatic metabolism However, the toxic status of *G. esculenta* has been difficult to establish due to variable toxin susceptibility within consumers [19]. Furthermore, it may be enhanced by age (elderly more susceptible) and/or some medical status (e.g., regular medication with isoniazid for tuberculosis; [9]). It was commonly believed by mushroom pickers that this species was not toxic since contradictory poisoning experience was reported. As a result, it is found as edible in mushroom lists proposed in France and Finland [16] and reported as gourmet food or as rejuvenating [9,13]. As popular belief is hard to change, its deliberate consumption was also reported in the Scandinavian countries. Development of local cooking practices that are supposed to partially detoxify this poisonous species by parboiling are used to justify such behaviour [9,22], so it is packaged and sold regularly on markets in these countries and can sicken consumers who are unaware of proper preparation procedures [9]. Although such processing inefficiency has been reported for a long time [8,19,23,24,25], the Finnish Food Authority only stated that *G. esculenta* is not to be eaten by pregnant and breastfeeding women and children because of residues of the toxin gyromitrin despite dried or cooked processing [22].

**Figure 2:**
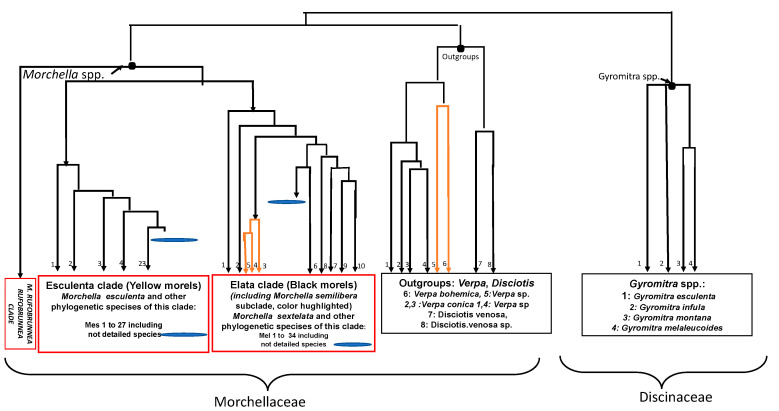
Presentation (schematic view) of a partial phylogenetic tree for positioning of some true and false morels (adapted from published data [15,17]; branches are respected but not the bootstrap values). *Morchella* species are classified morphologically into two main clades, black (Elata clade) and yellow (Esculenta clade), and the ascocarps within each species also differ in color nuances and shape [15]. *Verpa* species can show 99% similarity with *Disciotis venosa* and are members of the Morchellaceae family but are located in an outgroup [15,17]. *Gyromitra* species belong to another cluster, the Discinaceae family [15]. Mes for *M. esculenta;* Mel for *M. elata* [17].

Another false morel *Verpa bohemica* (called early false morel; Figure 1D), for which a synonym is *Ptychoverpa bohemica* [20], has an unclear edible/toxic status. It is absent from the lists of French edible mushrooms [16] but present in 4 out of 22 countries studied. It is commonly considered edible in France [26], in Switzerland (but not in Italy [16]) and in the French Alps where an ALS cluster was discovered [12]. Such differences between mushroom species present on official lists and mushrooms collected for consumption are common in Europe [27]. Moreover, having a wrinkled thimble-cap, it can be mistaken with *Morchella semilibera* (= *Mitrophora semilibera*), a true edible morel (Figure 1C). It is, however, a false morel phylogenetically located in an outgroup, outside of the clades of the constituent members of the *Morchella* genus (Figure 2; [15,17]). The rDNA sequence of *Verpa bohemica* forms a distinct subclade from the rest of species of the same genus *Verpa* ([15]; Figure 2).

Some poisonings with *Verpa bohemica* were reported from the NAMA’s case registry [28]. In the US, *Verpa bohemica* is usually regarded as suspect or toxic by many field guides (and some in Europe); even when it is reported as edible, it is generally not recommended for eating because it may cause gastrointestinal disorders in some people and caution is always recommended [29,30]. In fact, *V. bohemica* may cause variable reactions, including severe gastrointestinal upset and cerebellar syndrome with temporary loss of coordination in susceptible individuals [15,31]. However, it was surmised that the gastrointestinal and neurological symptoms reported after eating early morels, i.e., *Verpa bohemica* (in conspicuous quantities) might have always been misunderstood with the so-called “NS cerebellar syndrome” described thereafter [29]. Furthermore, another type of poisoning was reported with *Verpa bohemica* [32,33] as an inducer of Disulfiram-Like reactions (alcohol intolerance). *Verpa conica*, a similar species (Figure 2), has no identified toxicological status; however, it is not included in any list of edible mushrooms in Europe, except in France [16].

### 2.2. Poisonings from Crude, Cooked or Dried True Morels

Recent studies have described true morels of the genus Morchella [17]. This genus consists of the Esculenta Clade (yellow morels, Figure 1B), the Elata Clade (black morels, Figure 1C) and the Rufobrunnea Clade (blushing morels). Furthermore, the semifree capped morels (*M. semilibera*) were deeply nested within the Elata clade (black morels), contradicting an earlier opinion that semifree capped morels were the separate genus Mitrophora [17]. Actually, the polymorphic nature of the *Morchella* genus has contributed to its taxonomic ambiguity, so morphospecies names were replaced by the phylospecies names using codes starting with Mes (for the Esculenta clade) numbered from 1 to 27 or Mel (for the Elata clade) numbered from 1 to 34 [17] (Figure 2). Therefore, correct identification of true morels is difficult due to a great variety of morphotypes; thus, it can lead to misidentification with similar false morel species and thus can explain partly some of the poisonings described thereafter (especially at the dried state, for which initial shape is lost).

It has been known for decades that morels *Morchella* sp. are responsible for poisonings, causing either gastro-intestinal and/or neurological syndromes [32]. This has been reported by poison control centres (NAMA) in North America [28]. In Switzerland, it was reported [34] that among the 20 species most frequently involved in mushroom poisoning, 8 species are considered edible and comprise *Morchella esculenta*. Nausea and vomiting (which could also be attributed to haemolysin poisoning [35] with morels) were also reported in Portugal [36]. Morel species were also responsible of numerous poisonings in France [37].

These intoxications could be explained either by eating morels crude or insufficiently cooked or eating too much morels in one meal or in repeated meals. It has been known for years that morels are not edible if eaten crude; however, cooking at least 10 min in boiling water or more is efficient to ensure their edibility. This is the reason why they are classified in the conditionally edible mushrooms category [7]. Actually, when eaten crude or poorly cooked, a gastrointestinal syndrome was observed in the first 5 h after the meal, and it is attributed to some unknown haemolysins [25]. A neurological syndrome (NS) with cerebellar signs (tremor or dizziness/inebriation or unsteadiness/ataxia ± associated with gastrointestinal symptoms) associated with high consumption (up to 600 g) of known edible morels (*Morchella esculenta, Morchella conica, Morchella rotunda, Mitrophora semilibera, Morchella* spp.) was first described in France by [38,39]. The consumption of such high quantities can be done in one or several consecutive meals. It is the only positive factor, while the role of a preservation or cooking defect is not demonstrated or rejected [25]. It is a functional syndrome with long latency (mean of 12 h) with 6h for 94% of cases [25]; all these clinical signs were resolved within one day in 90% of cases. The syndrome occurs most frequently with fresh morels but also with dried-rehydrated mushrooms. In fact, it is an old recurrent poisoning not having been previously clinically described [39]. There is a great variability of susceptibility in consumers since not everyone is equally affected [40]. It is now included in the clinical classification of mushroom poisonings published recently [8]. The toxin(s) involved in this syndrome are unknown. Therefore, after fresh morels consumption, two poisoning syndromes, the GIT one (6 h) alone and the neurological long latency one (mean 12 h), were observed, which are due to poor cooking or over-consumption, respectively, whatever the cooking status [25].

Another additional hazardous potential comes from morels that are commercially available on the market in a dry state. Curiously, drying is believed to ensure morel edibility and this is a critical safety problem [21], since it is quoted everywhere on the internet that drying is an excellent way to completely eliminate or destroy toxins [21,28,32,39]. Rehydration is a key quality aspect for those dried products that have to be reconstituted before their consumption [41]. It was found that rehydration of the samples of *M. esculenta* at room temperature for about one hour absorbed less than 40% of the fresh product water content, while retaining 70% of the initial solids, and this rehydration ability reached a value similar to that of other vegetables. Therefore, we can advance the hypothesis that the loss of 30% of initial solids could explain some partial disappearing of toxicity of crude morels. The role of spore loss in this process should be also considered since these spores could contain an higher toxin amount than other tissues as observed in other mushroom fruiting bodies species [42].

## 3. Button Mushroom (and *Agaricus* spp.) and Agaritine 

### 3.1. Poisoning from Agaricus spp.

It is well established that *Agaricus* spp. including Portobello or Cremini *(Agaricus bisporus)* can be regularly responsible of a gastro-intestinal syndrome (diarrhoea, vomiting, cramps, nausea) [43] due to unknown poison classified as a gastrointestinal irritant [30] acting 1–2 h after consumption. These cases have been gathered from diverse poison control centres in Europe and the USA [28,32]. The corresponding species are *A. xanthodermus* and *A. moelleri* [7] in addition to those previously reported from NAMA’s registers [28]: *A. arvensis, A. augustus*, *A. californicus, A. hondensis, A. placomyces* and *A. praeclaresquamosus*. In Switzerland, it was reported [34] that among the 20 most frequently involved species in mushroom poisoning, 8 species are edible and comprise *Agaricus bisporus*. These edible species, in France, were also responsible for numerous poisonings [37]. The toxic compounds responsible for this acute toxicity are not known. However, the presence in the various *Agaricus* spp of a natural toxicant called agaritine and containing an hydrazinic moiety in its structure is worth considering, since toxicity of hydrazine derivatives, due to their high reactivity as chemical radicals, has been known for years [13,44,45]. Furthermore, curiously, agaritine poisoning is less and less mentioned in the recent literature except in two isolated studies published in 2014 [5,32] and an up to date is necessary.

### 3.2. Agaritine Presence in Mushrooms and Existence of Toxic Derivatives or Metabolites

Agaritine is a water-soluble γ-glutamyl-hydroxymethyl-phenyl hydrazine. It is specifically found associated to the Agaricaceae family and also present in some species of the genera *Leucoagaricus* and *Macrolepiota* [46]. It was detected at low levels in Shiitake (*Lentinus edodes* [32]). Different amounts were reported for 53 Agaricus species from the wild, and no less than 24 of the 53 species contained agaritine levels above 1000 mg/kg fresh weight [46]. The highest level was found in *A. elvensis* containing up to 10,000 mg/kg fresh weight. Seventeen species contained intermediate levels (125–1000 mg/kg) and twelve species were below 125 mg/kg. In Paris’ mushroom (another name of Button mushroom), agaritine is found at very variable concentrations from 50 to 1730 mg/kg of fresh weight [21] and at an average of 3040 mg/kg of spores [42]. It is thought to be produced in the vegetative hyphae in contact with the wheat straw compost, resulting respectively from the breakdown of lignin by the fungus and the diazotrophic activity of a bacterial commensal in the substratum and transfer to the fruiting bodies [47].

**Figure 3:**
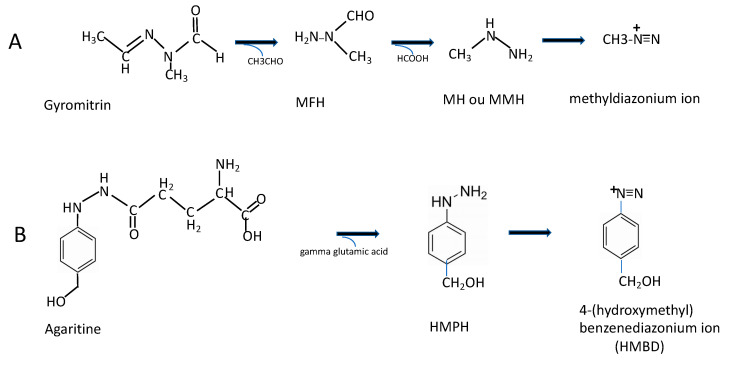
Schematic view of degradation of two hydrazinic toxins, Gyromitrin and Agaritine, into instablediazonium ions (from which reactive radicals are generated): (**A**) Gyromitrin: (acetaldehyde methylformylhydrazone)→N-methyl-N-formylhydrazine (MFH)→monomethylhydrazine (=MH or MMH). (**B**) Agaritine: (β-N-[γ-L-(+)- glutamyl]-4-hydroxymethylphenylhydrazine) →4-(hydroxymethyl)phenylhydrazine (HMPH)→ 4- (hydroxymethyl)benzenediazonium ion (HMBD).

It is oxygen sensitive. Contact with air, storage at ambient temperature, refrigeration or freeze-thaw cycles as well as cooking results in a decrease in concentration [21]. It was found that agaritine is comparatively heat-stable in *Agaricus* water extracts, since these retained 40–50% of the initial agaritine amount even after a 30–120 min incubation at 120 °C, contrary to pure agaritine which was heat-labile and easily decomposed at 100 °C [48]. However, agaritine is reduced 10-fold during the canning process [49]. It is stable at pH 6.8, but not at all at pH 1.2. Therefore, in the human stomach (pH range 2–3), it could be unstable, but the identity of its degradation products has not been studied [50]. Indeed, the presence of derivatives or metabolites associated to agaritine in the mushroom needs some clarification. The enzyme γ-glutamyltransferase was isolated from the button mushroom. It catalysed the hydrolysis of agaritine to glutamate and 4-(hydroxymethyl) phenylhydrazine (HMPH) [51,52]. However, the resulting HMPH was not always detected due to instability [53]. It has been also reported that agaritine in the button mushroom was hydrolysed to the 4-(hydroxymethyl) benzene-diazonium ion (HMBD) (to a 0.6 ppm level), by an enzyme system present in the button mushroom itself [45]. Furthermore, since a second diazonium ion is generated in acid extracts of *A. bisporus* from another precursor of unknown structure in the mushroom, commercial mushrooms may contain a compound capable of generating at least 20 μg/g (wet weight) of a diazonium ion under acidic conditions that mimic closely the conditions in the human stomach [45]. Another closely related nitrogen–nitrogen bond-containing compound (*p*-hydrazinobenzoic acid) was produced from β-N-[γ-L(+)-glutamyl] 4-(carboxy)phenylhydrazine (GCPH) present in button mushrooms and was shown to be carcinogenic through formation of phenyl radical [54]. Carcinogenicity of three other agaritine-related compounds was also reported [45]. Therefore, a number of metabolites are possibly produced from fresh button mushrooms and are associated to a potential carcinogenic toxicity when administered orally as salts into laboratory animals, especially mice [45]. In other species, evidence for the occurrence of the 4-hydroxy-benzene-diazonium ion in the extracts of *Agaricus xanthodermus* was also found [55]. The aldehyde agaritinal (β-N-[γ-glutamyl]-4-(formyl)phenylhydrazine) has also been demonstrated in significant amounts in *A. arvensis*, *A. augustus, A. campestris* and *A. macrosporus* mushrooms [32]. In vivo, the bioactivation of agaritine is believed to proceed via the initial loss of the γ-glutamyl group, catalysed by an internal (present into liver and kidney) γ-glutamyl transpeptidase, to release the free hydrazine HMPH, considered to be the pro-mutagen, which generates the ultimate mutagen, the HMBD ion, after enzyme oxidation [53]. The ultimate mutagen can bind to DNA and form adducts [53,56]. However, these ultimate compounds are chemical radicals that are very unstable and thus difficult to trace in the organism. A key piece of information on agaritine metabolism in mice was given by measuring agaritine concentration in mouse plasma and urine using specific LC/MS/MS [57]. Agaritine concentration peaked 20 min after oral administration to mice (4.0 and 40 mg/kg). The concentration gradually decreased and returned to the basal level in 100 min. The maximum concentration, the time to the maximum concentration and the half-life were 0.37 μg/mL plasma, 0.33 h and 0.71 h, respectively after administration of agaritine at 40 mg/kg body weight (b.w.). Results suggest that agaritine quickly metabolizes and disappears in the plasma, whereas DNA damage (evaluated by measurement of 8-hydroxy-2′–deoxyguanosine, a marker of oxidative stress) lasts for a long time (up to 11 days) after a single administration of agaritine to mice. Agaritine-COOH, which contains a carboxylic moiety instead of the hydroxymethyl one present in agaritine, is also present in *Agaricus* mushrooms, either *Agaricus blazei* or *Agaricus bisporus* [57]; when hydrolysed with the enzyme γ-glutamyl transferase, there is elimination of the γ-glutamyl moiety and production of the carcinogenic hydrazine derivative, *p*-hydrazinobenzoic acid reported above. Finally, the mutagenicities of *Agaricus* mushrooms are attributed to agaritine and agaritine-COOH, which can produce different free hydrazines, leading to the formation of diazonium ions and free radicals [57]. This metabolic trait is reminiscent of gyromitrin, the previous hydrazinic toxin studied above, which also generates instable diazonium ions, leading to free radicals and DNA disorders (Figure 3)

### 3.3. Feeding Studies

Some selected feeding studies gave further consistent results of mutagenicity/carcinogenicity of an agaritine-containing diet. An experimental balance feeding protocol of 12 h/12 h to switch from a diet consisting of cultivated baked button mushroom to a normal diet was used, and this allowed to report significant carcinogenic effects [58]. Chronic feeding of lyophilized button mushrooms also led to carcinogenicity [59]. A feeding study using the *lacI* transgenic Mouse Mutation Assay with Big Blue mice, which bear the bacterial *lacI* gene incorporated into a lambda bacteriophage shuttle vector, was stably inserted into the DNA of every mouse cell [60]. It provided further key information on genotoxicity of agaritine after isolation from genomic mouse DNA. Finally, the marker gene can be expressed in *E. coli* bacteria and mutations in the *lacI* gene detected with a colour test. Therefore, genotoxicity is quantified via determination of mutants frequency in the *lacI* gene, compared to controls. The agaritine content of the samples was determined by HPLC analysis and identification done using UV spectroscopy. During 15 weeks, female mice were given 1 of 3 mushroom diets, constituted of either agaritine fed daily 30 mg/kg b.w.(fresh mushrooms) or at 80 mg/kg b.w. (freeze-dried mushrooms) or, in the last case, of a crude agaritine mushroom extract at 120 mg/kg b.w. Significant genotoxic effects were observed only with the crude agaritine extracts. On the basis of previous work correlating carcinogenic potency in long-term feeding studies and mutagenic potency in the *lacI* test, these results were correlated with carcinogenic potency and combined with information on the average daily intake of *A. bisporus* to estimate the size of the carcinogenic risk posed by the consumption of mushrooms. The conclusion was that agaritine is a mild carcinogen. When translated to human consumption of button mushrooms, it was proposed that an intake of about 4 g of these mushrooms per day would be expected to contribute to a lifetime low cumulative cancer risk in humans of about 20 cases per 10^6^ individuals [60]. Weak genotoxicity was also confirmed later using ^14^C-Ring-labelled agaritine administered orally: the cumulative lifetime cancer risk of agaritine consumption in mushrooms was then estimated to lie at approximately 10^−5^ [61].

These assertions are in line with the estimated risk of 2–250 extra cancer cases during lifetime per million Nordic average consumers [32], updating their previous risk analysis [62]. The authors also underlined that the comprehensive set of data on raw and on processed button mushrooms and on the phenylhydrazine derivatives occurring in the mushroom indicate that this mushroom is carcinogenic in mice. However, no studies have demonstrated direct implication of agaritine or button mushroom consumption in any carcinogenic effects observed in humans, and agaritine is classified in the IARC group 3 (“not classifiable as to its carcinogenicity to humans”). However, it was stated that a carcinogenic risk for humans cannot be excluded, and the recommendations were not to eat button mushrooms in large amounts and to use proper processing of the fresh or dried rehydrated mushroom to reduce the amount of potentially carcinogenic constituents [32].

## 4. Discussion

The present paper focuses on some hydrazinic toxin-containing mushrooms from the genera *Gyromitra*, *Verpa*, *Morchella* and *Agaricus*. Due to a very high worldwide level of production and consumption, given the potential safety issue, proper processing through relevant recommendations is needed. 

For the FDA, *Gyromitra* spp., *Helvella* spp., and *V. bohemica* are lumped together as toxic false morels [63]. USA regulatory controls include FDA Import Alerts for true morels coming from France since they were known to be possibly mixed with *Gyromitra esculenta* and *Verpa bohemica.* In case of non-compliance of the shipment, this results in refusal of admission of dried and canned morels as US import and failure to obtain custom clearance for firms quoted on a red list [64]. In the past, the toxin(s) responsible of *V. bohemica* poisoning was thought to be gyromitrin [63]. This is quite possible for two reasons: (i) similar gastrointestinal symptoms are observed, at low dosage, during *Gyromitra* spp. poisoning [8] and (ii) such a poisoning depends also on personal metabolic susceptibility [29], as for *Gyromitra* spp. [8]. Therefore, edibility of *V. bohemica* is questionable as was that of *Gyromitra esculenta* for years. Concerning “haemolytic” poisoning due to true morels if eaten crude, it is reminiscent of what is known about some vegetables [35] or Fungi [65] and the association with generally heat-labile haemolysins.

An agaritine risk assessment analysis was done in 2010 by an Australian team [66]. They concluded that agaritine from consumption of cultivated *A. bisporus* mushrooms poses no known toxicological risk to healthy humans. However, this assertion was mainly based on studies on patient groups having different diseases, in clinical trials lasting up to one year and using other mushrooms species than Button mushrooms, so it is not very relevant for the estimation of potential cancer risk resulting from consumption of Button mushrooms. The rodent experimental feeding studies were also criticized by the same team [66]. However, on the contrary, a more recent and exhaustive risk assessment study, concluded the button mushroom is carcinogenic in mice [32], and that extrapolation in human is possible [32] as presented above. The presence of unknown metabolites other than those described here, in the mushroom, also complicates the data, since they could play a role in poisoning [67].

In France, an average consumption of 17.4 and 12.7 g of mushrooms per day per person is estimated for adults and children, respectively, according to the second Individual National Food Consumption study [68]. In Nordic countries, a lower range from 0.6 kg to 2.1 kg/person/year has been reported for consumption of Button mushrooms. However, if these values are below the 100 g per week tolerated intake proposed previously for morels [32], the group of regular and/or heavy mushrooms consumers must be taken into consideration. As estimated, 5% of the consumers are heavy consumers, eating more than five times the median intake, while the 0.1% of the heaviest consumers eat up to thirty times more than the median intake [32]. These high consumers are more exposed to agaritine poisoning (and morel poisoning) than other groups of sporadic consumers. Furthermore, it was not taken into account that these edible mushrooms, apart from their very high culinary interest, have also a medicinal interest and also are commonly consumed as nutraceuticals or functional foods [17,69,70,71,72,73].

The present literature analysis was then done only to support a claim for a need for precautions of safe use for false or true morels and button mushrooms. Obviously, as *Agaricus bisporus* is one of the most consumed mushrooms around the world, it would be a real revolution if it turned out that it was no longer considered edible. It would nevertheless be good to remember that it should not be considered an everyday food and that as a matter of prudence, it should not become one, especially when eaten raw.

Before releasing marketed mushroom products, the food industry (food supplements included) has to deliver them in compliance with hygienic and hazardous analysis (HACCP) used for Food hygiene policy (Available online: https://ec.europa.eu/food/safety/biosafety/food_hygiene_en). Therefore, our present analysis of critical safety points paves the way for reinforcing these safe hygienic and HACCP practices used by the mushroom industry. All the successive technical operations for the processing of mushroom species to be put on the market should be described and analysed through a dedicated Good Hygiene Practices Guide, which is still missing [74]. Some data are available in a guide dedicated to dehydrated legumes (including mushrooms) and aromatic plants [75], but it is insufficient, and no other corresponding guide is available so far [76]. For Button mushrooms, a threshold value for the diverse marketed preparations is needed and should be established under a Food Safety Agency control; then, this value could be used as a critical safety point in HACCP process analysis done before releasing mushroom products to the market. Such a safety approach is needed to secure the safe consumption of button mushrooms, especially for raw fresh products designed for eating raw. Fresh button mushrooms are commonly used as a salad in a lot of countries in Europe and in other parts of the world including USA. Here, a role of CTCPA [77], which assists the Food industry [78], is necessary (especially by emitting new appropriate decisions [79]).

## 5. Conclusions and Recommendations

In conclusion, a warning needs to be issued (i) for false morels which are inedible and (ii) for crude fresh or dried, true morels or button mushrooms, which must not be consumed in any case without an obligatory efficient cooking in boiling water for at least 10 min or more, discarding the water before use. Ordinary freezing and subsequent thawing (but not freeze-drying) is known to reduce the content of hydrazinic compounds or haemolysins in the mushroom. Furthermore, proper processing should be recommended before eating air-dried products with a rehydration step and, as with fresh products, cooking in boiling water, which is then eliminated, is recommended. Before consumption of canned or jar mushrooms, soaking water also needs to be discarded since it reduces the amount of hydrosoluble toxins such as agaritine and other hydrazinic or water-soluble or heat-labile compounds such as haemolysins. For button mushrooms, food-processing companies should use an agaritine threshold level value for safety processing of their products, especially if marketed in a raw state. For all these mushrooms, the amount consumed should be limited, at most, to a tolerated intake of 100 g fresh (or fresh equivalent if dried or freeze-dried mushrooms are used) per week in one or more meals. For air-dried products, we recommend writing on the labels that these dried morels or button mushrooms must be consumed well-cooked after rehydration and elimination of cooking water.

The main safety alert is not to consume any raw or poorly cooked fresh or dried morels or button mushrooms, and this should be notified quickly, especially to high consumers and susceptible population such as women desiring pregnancy. According to the new behaviour diet, long term effects of a regular ingestion should be evaluated using objective DNA or neural damages criteria and other points underlined in the present paper.
