# Correction: Lagrange, E.; Vernoux, J.-P. Warning on False or True Morels and Button Mushrooms with Potential Toxicity Linked to Hydrazinic Toxins: An Update. *Toxins* 2020, *12*, 482

**DOI:** 10.3390/toxins14050345

**Published:** 2022-05-16

**Authors:** Emmeline Lagrange, Jean-Paul Vernoux

**Affiliations:** 1 EFSN, Pôle de Neurologie, CHUGA, Grenoble University Hospital, 38000 Grenoble, France; elagrange@chu-grenoble.fr; 2 Unité de Recherche Aliments Bioprocédés Toxicologie Environnements (ABTE) EA 4651, Normandie University, UNICAEN, 14000 Caen, France

The authors wish to make corrections to their paper [1], due to the recent retraction of the paper by Phanpadith et al., 2019 [2], referenced as reference 15 in the published paper. Anand et al., 2013 [3], and Du et al., 2015 [4], are cited thereafter.

On page 2, in the third sentence of the second paragraph, reference 15 [2] is replaced by Anand et al., 2013 [3]. On page 3, bottom line only, reference 15 [2] is replaced by Anand et al., 2013 [3]. On page 4, in the caption of Figure 2, the two mentions of reference 15 [2] are removed. In Section 2.2, the first two citations of reference 15 are removed; the third is replaced by Du et al., 2015 [4], and the sentence “A novel lineage was also described in the Esculenta clade [15]” is entirely removed. In the section References, page 11, details about reference 15 [2] are also removed entirely. The order of all other references is adjusted accordingly.

The changes do not affect the scientific results. The manuscript will be updated and the original will remain online on the article webpage. We apologize for any inconvenience caused to the readers.
